# Leukemia Cutis—The Current View on Pathogenesis, Diagnosis, and Treatment

**DOI:** 10.3390/cancers15225393

**Published:** 2023-11-13

**Authors:** Ewa Robak, Marcin Braun, Tadeusz Robak

**Affiliations:** 1 Department of Dermatology, Medical University of Lodz, 90-647 Lodz, Poland; ewarobak@onet.eu; 2 Department of Pathology, Medical University of Lodz, 92-213 Lodz, Poland; marcin.braun@umed.lodz.pl; 3 Department of Hematology, Medical University of Lodz, 93-510 Lodz, Poland; 4 Department of General Hematology, Copernicus Memorial Hospital, 93-510 Lodz, Poland

**Keywords:** acute leukemia, chronic leukemia, cutis, diagnosis, pathogenesis, prolympchocytic leukemia, Richter transformation, skin lesions, treatment

## Abstract

**Simple Summary:**

Leukemia cutis occurs in different types of leukemia, most commonly in chronic lymphocytic leukemia and acute myeloid leukemia. Its varied clinical appearance makes it difficult to differentiate from other skin lesions. The leukemic skin changes are localized or disseminated and can be located in any site of the body. The diagnosis is mainly based on the clinical characteristics and histopathologic, as well as immunophenotypic features of the skin lesions. The treatment of leukemia cutis depends on the specific diagnosis of hematologic malignancy and is aimed at eradicating the primary underlying disease. Local irradiation for skin lesions is sometimes useful for palliative treatment.

**Abstract:**

Leukemia cutis (LC) is defined as the leukemic infiltration of the epidermis, the dermis, and the subcutaneous tissue. Leukemia cutis may follow or occur simultaneously with the diagnosis of systemic leukemia. However, cutaneous lesions are occasionally diagnosed as the primary manifestation of leukemia. Leukemic skin infiltrations demonstrate considerable variation regarding a number of changes, distribution, and morphology. The highest incidence of LC is observed in chronic lymphocytic leukemia, monocytic and myelomonocytic acute myeloid leukemia, and T-cell lineage leukemia. Although the pathogenic mechanism of the invasion of leukemic cells into the skin is not well understood, chemokine receptors and adhesion molecules as well as the genetic characteristics of leukemia are thought to play a role. Leukemic skin lesions may be localized or disseminated and may occur alone or in combination on any site of the skin, most frequently in the trunk and extremities. The most common clinical presentations of leukemia cutis are papules, nodules, macules, plaques, and ulcers. In most patients, the complete or partial resolution of cutaneous infiltrations occurs simultaneously with hematologic remission. However, in patients with resistant disease or recurrent skin infiltration, local radiotherapy can be used. This review presents recent data on the pathogenesis, diagnosis, and treatment of leukemic skin involvement in different types of leukemia.

## 1. Introduction

Leukemia cutis (LC) is manifested as clinically demonstrated skin infiltration via neoplastic leukocytes or their precursors into the epidermis, dermis, or subcutaneous tissue [1,2,3,4]. It is a relatively rare symptom observed usually in more advanced stages of the disease. The frequency of LC varies from 2% to 30%, depending on the diagnosis of primary leukemia [2,5,6,7,8]. Leukemia cutis may follow or occur simultaneously with the diagnosis of systemic leukemia. However, LC is occasionally diagnosed as the primary manifestation of leukemia. All subtypes of leukemia can infiltrate the skin. However, LC is most commonly observed in children with congenital leukemia, ranging from 25% to 30% of cases [9]. Generally, the highest incidence of LC has been noted in acute myeloid leukemia (AML) of, monocytic and myelomonocytic FAB (French, American, British) subtypes, chronic lymphocytic leukemia (CLL), chronic lymphocytic leukemia (CLL), and the T-cell leukemias [6,10,11]. Leukemia cutis frequently indicates an advanced disease with additional sites of extramedullary involvement, which is strongly associated with poor prognosis. Larger studies of leukemia cutis are presented in Table 1.

In 55% to 77% of cases with LC, skin lesions occur in patients already diagnosed with leukemia [7,8]. However, leukemic skin infiltrations are seen at leukemia diagnosis in 23% to 44% of cases and can precede diagnosis in peripheral blood (PB) and/or bone marrow (BM) by several months or years in 2% to 3% of cases [2]. Cutaneous involvement usually indicates advanced disease, and extramedullary involvement in other sites is common.

Risk factors for the development of LC are generally the same as those for systemic leukemia, including exposure to benzene, ionizing radiation, alkylating agents, and viruses. In patients with LC, prognosis is generally poor [3,16,17]. However, it can be improved by appropriate treatment with novel drugs.

This review provides an overview of the pathogenesis, diagnosis, and treatment of leukemic skin involvement in different types of leukemia.

## 2. Pathogenesis

The pathogenic mechanism of leukemic skin involvement is not well known. However, chemokine receptors and adhesion molecules, as well as the genetic characteristics of leukemia, are believed to play a role [7]. In particular, adhesion molecules such as chemokine integrin may be involved in the migration of leukemic cells into the skin via skin-selective homing processes [7,18,19,20]. In addition, important roles may be played by chemokine receptors and adhesion molecules. For example, the cutaneous leucocyte-associated antigen (CLA) receptor and CC chemokine receptor 4 (*CCR4*) on the leukemic cells may interact with E-selectin and/or TARC (thymus- and activation-regulated chemokine/CCL17 (CC chemokine ligand 17) on the dermal post-capillary venules. This process may stimulate the movement and binding of leukemic cells into the dermis; in addition, such migration of leukemic cells into the dermis may also be stimulated via the interaction between integrins and endothelial-bound chemokines. Some observations indicate that the infiltration of leukemic cells is more likely to occur in places with previous skin infections or inflammation [21].

## 3. Diagnosis

A diagnosis of LC requires an evaluation of clinical features, morphology, histopathology, and immunophenotyping [7,8,21]. While LC is most commonly characterized as nodules, plaques, ulcers, vesicles, and swellings [2,13,18,19], unusual clinical manifestations are occasionally observed, including erosions, ulcerations, and desquamation [2,22,23]. Moreover, leukemic vasculitis has also been noted as an unusual manifestation of LC, while it occurs mostly in patients with acute leukemia with myelomonocytic or monocytic features [14,22,24]. Skin changes are mainly located in the trunk, extremities, and face [13,19,21]. Widespread petechiae-like eruptions secondary to LC have also been rarely described [25]. Although skin lesions are usually generalized, some solitary, clustered, or dispersed lesions have also been observed [2]. In rare cases, the distributions of LC can include sites of herpetic lesions, intravenous catheters, lips, trauma, and recent surgeries [7,26,27].

Some authors indicate that generalized LC occurs mainly in acute leukemias, and single lesions are observed mainly in less aggressive hematologic malignancies [2]. However, most studies have found no correlation between the location and distribution of skin leukemic infiltration with regard to the specific type of disease [15,28]. Leukemic lesions can be violaceous or brick-red to skin colored. Leukemia cutis may also exist as diffuse purpura, particularly in infants, such as blueberry muffin syndrome. In most patients, the skin lesions are asymptomatic, but occasionally, pain or pruritus may be present [5,29]. The development of LC is more likely to be rapid in acute leukemias but more gradual in chronic leukemias [30]. The appearance of the skin lesions in LC is nonspecific, which makes it difficult to clinically differentiate from other skin lesions [13,31,32].

The diagnosis of LC requires the evaluation of biopsy specimens with immunohistochemical staining, with the diagnosis confirmed by determining the expression of characteristic cell surface markers [33]. Immunohistochemical staining remains essential for distinguishing reactive from neoplastic infiltrates, as LC can clinically mimic the reactive lesions. Skin biopsy shows nodular or diffuse infiltrations with leukemic cells in the dermis and/or subcutaneous tissue. In most cases, the leukemic infiltrations do not involve the epidermis and upper dermis, known as the “grenz zone”. However, in T-cell leukemias, epidermotropism is a common event.

The differential diagnosis of LC includes neoplastic, inflammatory, and infectious skin lesions [34,35]. Cutaneous paraneoplastic disorders are defined as one group of nonleukemic cutaneous leukemias that comprise the cutaneous paraneoplastic disorders. Such skin changes have been noted in more than 40% of leukemia patients and are more common than LC. The condition manifests as petechiae/purpura caused by thrombocytopenia, neutrophilic dermatoses such as pyoderma gangrenosum and Sweet’s syndrome, as well as leukocytoclastic vasculitis [5,36]. Other studies have also reported the occurrence of opportunistic infections, including disseminated candidiasis and herpes zoster. Any erythematous bright-red or red-brown plaques in LC should be differentiated with erythema exudativum multiforme, panniculitis, or mycosis fungoides [2,7]. Infiltrated erythema and flat nodules should be distinguished from erythema nodosum. Hemorrhagic or purpuric nodules and plaques on the trunk or the lower legs should be differentiated with vasculitis allergica and Kaposi sarcoma. Macular or maculopapular exanthems may resemble pityriasis rosea, viral exanthems, or drug eruptions.

## 4. Prognosis

In patients with LC, prognosis depends on the leukemia type and advancement of the disease. As LC coexists with systemic leukemic involvement, the prognosis is rather poor, especially in patients with other extramedullary infiltrations and in cases when LC is diagnosed with advanced leukemia, resistant to previous therapies. A worse prognosis is also observed in AML, T-cell prolymphocytic leukemia (T-PLL), and Richter’s syndrome (RS) [6,37,38,39]. In a recent study, the median survival time of patients with LC was 7.2 months, with no statistically significant difference between different types of leukemia [5,13]. In another study by Yook et al., 93% of patients died within 10 months after diagnosis [3]. Similar results were reported by Su et al., where 88% of patients with LC died within one year from diagnosis of LC in acute lymphoblastic leukemia (ALL), AML, CLL, and other leukemias [1].

## 5. Treatment

Leukemia cutis is a local manifestation of an underlying systemic disease and should be treated with systemic therapy appropriate to the specific subtype of leukemia. In most patients, hematologic remission occurs simultaneously with complete or partial response of cutaneous infiltrations. However, in patients with resistant LC or recurrent skin infiltration, local radiotherapy can be used [34,40]. Recently, simultaneous integrated boost with helical arc radiotherapy of total skin (HEARTS) has been proposed to treat cutaneous manifestations in the treatment of refractory cutaneous leukemia [41].

## 6. Characteristics of Leukemia Cutis in Different Subtypes of Leukemia

### 6.1. Chronic Lymphocytic Leukemia

Chronic lymphocytic leukemia or small lymphocytic lymphoma (CLL/SLL) is the most common adult leukemia in Western countries, with an estimated 20,050 new cases in 2022 in the United States [42,43]. It is a clinically and biologically heterogeneous disease with a variable clinical course. Only a minority of patients have an aggressive disease at diagnosis requiring treatment. Most patients have indolent disease and rarely require intervention [44].

Cutaneous symptoms in CLL have been described in 4–20% of CLL [45]. Most commonly, skin changes are secondary, nonmalignant lesions such as purpura, pruritus, urticaria, erythroderma, pyoderma gangrenosum, cutaneous vasculitis, and Sweet’s syndrome [2]. However, histologically confirmed LC is a rare event in CLL and most frequently occurs in patients with Richter transformation. Typically, skin LC occurs late in the natural history of the disease, most commonly after multiple lines of treatment and relapses. In contrast, CLL infiltration in the skin is only occasionally the first manifestation of the disease [38,46,47,48,49,50,51,52,53]. In some cases, leukemic skin infiltrations develop at the previously affected healed sites of *Borrelia burgdorferi*, herpes zoster, or herpes simplex [51,54,55].

**Figure 1:**
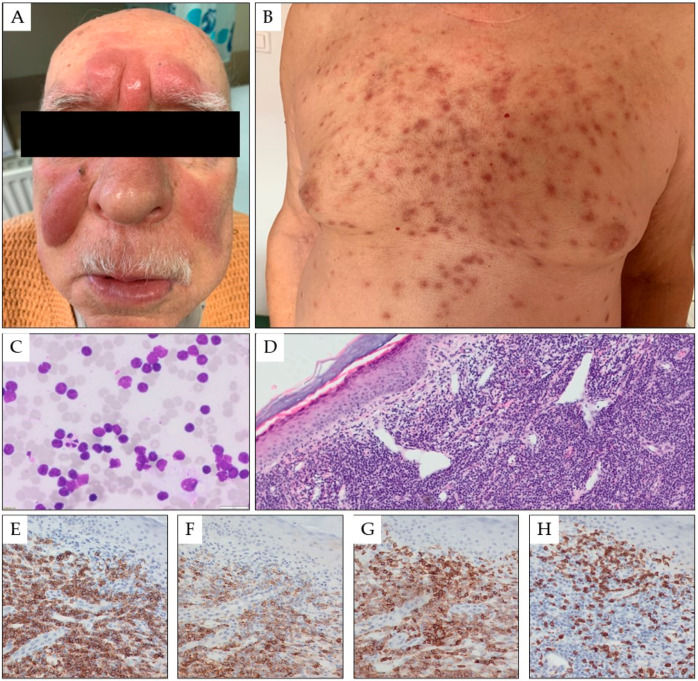
Multiple red-brown tumors and nodules on the skin of the central surface of the forehead and the cheeks of a 78-year-old male patient with CLL in the form of symmetrical nodular infiltrates (**A**). Moreover, numerous, scattered red-blue papules and nodules with a hemorrhagic reaction are present on the torso (**B**). Fine needle biopsy of the forehead tumor (1000× magnification) (**C**) and biopsy of the skin torso infiltration in hematoxylin and eosin staining (25× magnification, panel (**D**)), and in immunohistochemistry for CD20 (200× magnification, panel (**E**)), CD23 (200× magnification, panel (**F**)), CD5 (200× magnification, panel (**G**)), and CD3 (200× magnification, panel (**H**)).

Skin infiltration with B-CLL/SLL can manifest as solitary, grouped, or generalized papules, plaques, nodules, or large tumors (Figure 1). The most common skin site manifestations are the head and neck (34% of lesions) and trunk or extremities (27%) [56]. Immunohistochemistry and genetic studies are useful in proper diagnosis. In CLL, leukemic cells are small, with mature-appearing cells that show perivascular, periadnexal, or nodular distribution. Skin infiltrates via CLL/SLL most commonly exhibit a classic immunophetype: CD20+, CD3-, CD5+, CD23+, and cyclin D1-. Three main histologic patterns of CLL skin infiltrations have been reported, including perivascular and periadnexal lymphoid infiltration around vessels/adnexal structures, with a nodular, diffuse, and band-like pattern [38].

**Figure 2:**
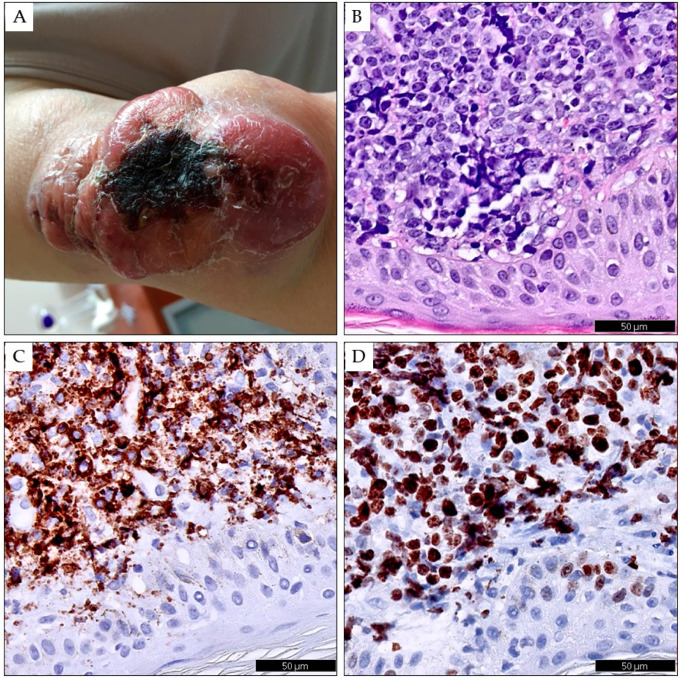
Richter transformation in the skin of the left upper limb in a 78-year-old female patient with CLL lasting four years. The skin shows a conglomerate of merging red-blue tumors of various sizes with a necrotic scab in the center (**A**). Skin biopsy showing infiltration by diffuse large B-cell lymphoma in hematoxylin and eosin staining (**B**), in immunohistochemistry for CD20 (**C**), and Ki67 (**D**). Digital scans were obtained using Phillips IntelliSite UltraFast Scanner. (Philips, Amsterdam, The Netherlands.)

In contrast to other leukemias, the prognosis in most CLL/SLL patients is not affected by skin involvement [38,45]. However, it is unfavorable in the RT of CLL with a specific infiltration of the skin and if LC is diagnosed later in the disease course (Figure 2) [50,57,58]. Cerroni et al. analyzed the clinical, histopathologic, immunophenotypic, and molecular features of 42 patients with SLL/CLL skin involvement [38]. The mean duration of CLL before skin manifestations was 39 months (range 0 to 142 months). In seven patients (16.7%), the skin lesions represented the first sign of CLL. Follow-up data could be obtained from 31 patients. Two patients with RT died after five and eight months. The five-year survival rate of the patients was 66.6%.

Leukemia cutis of CLL can be treated with conventional immunochemotherapy and, more recently, with Bruton’s tyrosine kinase inhibitors (ibrutinib, acalabrutinib, and zanubrutinib) or B cell lymphoma 2 (BCL-2) inhibitor venetoclax [59,60,61]. However, local therapy, including radiation therapy, excision, intralesional steroids, ultraviolet light B, and electrochemotherapy can be useful in local control of the skin lesions [62,63].

### 6.2. Hairy Cell Leukemia

One subtype of B-cell indolent lymphoid leukemia is hairy cell leukemia or HCL. It typically manifests as infiltration of mature lymphocytes into various organs and can typically be observed as hairy projections. It can be found in the peripheral blood (PB), bone marrow (BM), and spleen. HCL often results in splenomegaly, pancytopenia, and a higher susceptibility to infection [64]. HCL cells are positive for inter alia CD19, CD20, and CD22 B cell antigens and the CD11c, CD25, CD103, CD123, and TRAP HCL-specific antigens. The cells also demonstrate intense CD200 expression [65]. A recent study found *BRAF*-V600E to be an oncogenic mutation specific for classic HCL [66].

Around 10% of HCL patients develop skin lesions. These changes are generally related to autoimmune processes, infections, or secondary cutaneous neoplasms [67]. However, it is very rare that the skin undergoes leukemic infiltration via HCL cells [68,69,70]. A study of 600 HCL patients identified LC in 48 (8%) patients, but only eight of these (1.6%) were confirmed histologically [71]. LC has usually been diagnosed at the presentation of HCL; however, it has also been noted in the course of the disease [71,72,73,74,75,76]. In cases of HCL, LC infiltration is usually characterized by papules and plaques, as well as violaceous to red-brown or flesh-colored nodules. In some cases, the lesions may be found on a single side, and in others, they can be generalized over the body [8]. All patients suspected of leukemic skin infiltration should be recommended for skin biopsy and immunophenotyping. In addition, any patients demonstrating such skin changes should be further tested for clinical and laboratory signs of HCL, including BM and PB immunophenotyping and molecular testing [7,8,61].

In HCL patients, LC responds well to purine analog treatment for leukemia and can be successfully treated with cladribine or pentostatin. Treatment with cladribine also typically resulted in the disappearance of cutaneous infiltrates, with the complete resolution of the skin lesions. More recently, approaches based on treatment with BRAF inhibitors, including vemurafenib and dabrafenib, have been used in refractory or relapsed patients [77].

### 6.3. T Cell Prolymphocytic Leukemia

**Figure 3:**
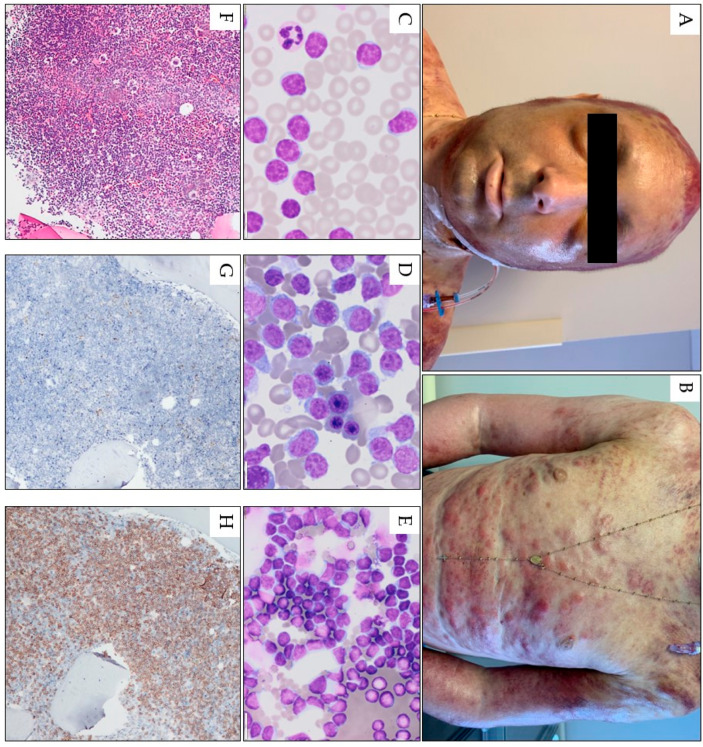
Pink-red papules, nodules, and tumors scattered on the face torso, and upper limbs in a 32-year-old male with T-PLL. A confluent erythema with a hemorrhagic reaction is also visible in the area of the scalp. Generalized skin edema, scattered petechiae, and conjunctivitis are also visible (**A**,**B**). Peripheral blood smear (1000× magnification) (**C**) and bone marrow smear (1000× magnification) (**D**) and fine-needle skin aspiration smear (1000× magnification) (**E**) show numerous atypical prolymphocytes. In bone marrow trephine, a homogenous infiltrate by CD3-positive, TdT-negative prolymphocytes is seen; hematoxylin and eosin staining image under 100× magnification (**F**) and in immunohistochemistry for TDT (100× magnification, (**G**)), and CD3 (100× magnification, (**H**)).

The most common type of mature T-cell leukemia is T-cell prolymphocytic leukemia (T-PLL). Nevertheless, it has a low prevalence: in adults, it accounts for only 2% of small lymphocytic leukemias [78]. Most cases of T-PLL are, unfortunately, resistant to conventional chemotherapy. In suitable patients, a combination of anti-CD52 monoclonal antibody alemtuzumab followed by consolidation with allogeneic hematopoietic stem cell transplantation is currently the most effective treatment; however, the median survival is only 17 to 33 months in patients treated with alemtuzumab alone and 48 months in those with allogeneic stem cell transplantation [79,80]. Around 25–30% of T-PLL patients develop cutaneous involvement, typically at presentation [81,82,83,84]. Although the skin lesions mainly present as retiform hyperpigmented macules and patches, they can also be found as skin nodules, erythroderma, symmetrical distributed petechia or purpura, and facial eruptions (Figure 3) [82,83,85,86]. In some cases, conjunctival involvement has been reported with periorbital petechiae (Figure 3) [83,85,87,88].

Other manifestations include diffuse infiltrative erythema and nodules and exfoliative dermatitis over the whole body. The most common facial involvement of LC is edema [16,83,87,88,89]. In most patients, skin biopsy shows extensive monotonous infiltration of prolymphocytes positive for T-cell immunohistochemical markers, including CD2, CD3, CD5, and CD7, while negative for TdT, CD1a, and variably positive for CD4 and CD8 (Figure 3) [90].

In a study of 25 TPLL patients by Hsi et al., eight (32%) demonstrated cutaneous manifestations, presenting as rash, purpura, papules, and ulcers; the skin biopsies showed leukemic lesions with small to medium-sized, irregular, perivascular, and periadnexal lymphoid infiltrates without epidermotropism. Cutaneous involvement via T-PLL was often associated with significant peripheral blood involvement [91]. Shumilov et al. observed three relapsed patients with T-PLL, including one with skin manifestations [92]. Only one of these patients relapsed with skin manifestations. Wasitudin et al. described another patient with diffuse generalized skin lesions, rash, and anasarca [82], and Matutes et al. noted the presence of skin lesions in 27% of 78 patients with T-PLL [93].

### 6.4. Acute Lymphoblastic Leukemia

Acute lymphoblastic leukemia is the second most common acute leukemia in adults, with an estimated 6660 new cases in 2022 in the United States [42]. Acute lymphoblastic leukemia most commonly affects children younger than 15 years old and adults older than 50 years old [94,95]. In adults, ALL contains 20% of all leukemias [96]. The disease is divided into two main types: B-cell ALL and T-cell ALL. The T-cell ALL subtype is less common than B-cell ALL and is observed in around 20–25% of all ALL cases. Dose-intensification systemic treatment has led to significant progress in outcomes for children with ALL. However, the prognosis for the elderly remains very poor, with only 30–40% long-term remission in adult patients with ALL [97].

Leukemia cutis rarely presents in patients with ALL and may be seen in only 1% of cases [7]. Skin involvement has been reported in 1% to 3% of ALL patients [17,98,99]. In most patients with ALL, LC appears as single or multiple red-to-violaceous papules, nodules, and plaques [1,25,26,99,100,101,102]. Occasional oval or annular scaling red patches on buttocks, thighs, and back waist are observed. Systemic chemotherapy and stem cell transplant can be the primary treatment in some patients. However, radiation therapy can be used in patients with limited lesions, especially if they are not candidates for chemotherapy and stem cell transplant. Leukemia cutis is usually associated with T-ALL but occurs only occasionally in B-ALL [103].

Only three cases of LC associated with B-ALL have been reported recently [103,104,105]. One case was characterized by an asymptomatic, solitary, dome-shaped, indurated nodule on the left cheek [104], and the second by an erythematous, indurated, purplish nodule on the nose, with larger, erythematous nodules on the forehead and forearm [105]. Finally, the third manifested as a non-healing leg ulcer and patches on the left arm, face, and bilateral legs [103]. In all patients, immunohistochemical staining found the infiltrations involving the skin to be consistent with B-ALL.

More cases of LC were reported in T-ALL [5,25,106,107,108,109,110]. Leukemia cutis in T-ALL may present with erythematous papules, annular plaques, and petechiae-like eruptions [23,25,102]. In T-ALL, leukemic infiltration appears in the dermis and the subcutaneous tissue; however, in contrast to primary cutaneous T-cell lymphoma, the epidermis is uninvolved. Leukemic cells typically display T lineage-specific antigens, including CD3, CD4, or CD8, and one or more precursor immature T-cell antigens, including TdT, CD99, CD34, and CD1a [111]. The prognosis of LC in ALL is rather poor. However, in rare cases, longer survival was observed, especially after treatment with a combination of chemotherapy and allogeneic stem cell transplantation [25,110,112].

### 6.5. Acute Myeloid Leukemia

Acute myeloid leukemia is the second most common leukemia in adults, with 20,050 new cases in 2022 in the United States [42]. In total, 10–15% of AML cases were reported to have involvement of skin; this was most commonly observed in the myelomonocytic (AMML) and monocytic (MoAML) subtypes and with a higher prevalence in congenital leukemia (25–30%) [2,7,15,18,21,39]. Aleukemic LC is observed in 7% of cases with AML [113]. While LC most commonly develops in patients with an established diagnosis of AML, it is occasionally seen before a diagnosis of systemic AML [114]. AML LC can be associated with extramedullary leukemic involvement at other sites, most commonly the central nervous system (17%) [34]. In addition, gingival hyperplasia can be observed, particularly in AMML (42%) and MoAL (55%) [115]. Some gene abnormalities are associated with LC presentation of AML, including numerical abnormalities of chromosome 8, translocation (8;21) (q22;q22), and inversion (16) (p13;q22) [6,12,113,116,117,118].

**Figure 4:**
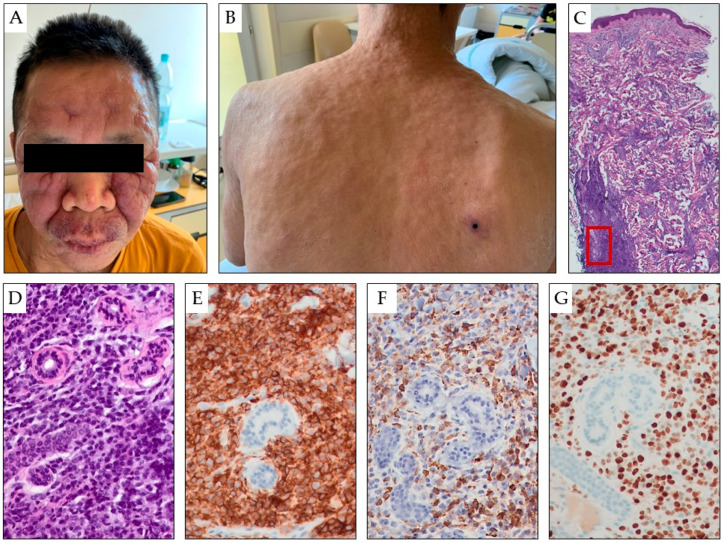
Asymptomatic, multiple, variable-sized, erythematous tumors and papules are present on the face (**A**) and torso (**B**) of a 49-year-old patient with AML. The entire skin was thickened and infiltrated, with numerous red-blue tumors and nodules scattered over the entire surface. A particularly large, hard infiltrate is visible on the facial skin, where erythematous and exfoliative foci are also present on the basis of the nodular infiltrate (**A**,**B**). Biopsy of the skin showing infiltration by myeloblasts (hematoxylin and eosin staining in panels (**C**,**D**) under 25× and 200× magnification, respectively), which are positive for CD45 (LCA, 200× magnification, panel (**E**)), myeloperoxidase (200× magnification, panel (**F**)), and Ki-67 proliferation marker (200× magnification, panel (**G**)). The red box indicates area shown under larger magnification in panels (**D**–**G**).

The most frequent lesions in AML LC are erythematous or violaceous papules and nodules, which are observed in 60% of AML LC patients (Figure 4). Infiltrated plaques, a generalized cutaneous eruption, and erythroderma are also noted [39]. Myeloid leukemia cells also involve the dermis and subcutis in a diffuse pattern. A skin biopsy serves as the gold standard for diagnosis. Histopathologically, infiltrates of blasts with varying intensity, positive for at least two myeloid markers, are essential criteria for establishing the diagnosis. Extensive immunophenotyping is clinically important to differentiate AML LC from blastic plasmacytoid dendritic cell neoplasm—the latter being positive specifically for TCF4, CD303, TCL1, and less strongly positive for CD4, CD56, and CD123, while negative for CD34, CD117, CD15, and myeloperoxidase.

There are no specific treatment options for patients with AML LC. Initial treatment is determined by factors such as age, performance status, cytogenetics, and molecular markers [119,120,121,122,123]. The standard induction regimen for younger, fit patients is based on a combination of cytarabine and anthracycline, known as the 7 + 3 regimen. Various molecular markers, including mutations in *FLT3*, *NPM1*, *CEBPA*, and *IDH* mutations, can influence treatment decisions. The incorporation of targeted drugs, such as midostaurin, in *FLT3*-mutated patients has shown improved prognosis, but its impact on LC in AML patients remains unknown.

Simultaneous integrated boost (SIB)–helical arc radiotherapy of total skin (HEARTS), a modified version of helical irradiation of total skin (HITS) therapy, has been used to treat AML in some patients with disseminated LC [41]. Wang et al. reported a 5-year survival rate of 8.6% among 62 patients with AML and LC; this value was significantly lower than that observed for 186 matched AML patients without LC (28.3%) [119]. In addition to conventional chemotherapy, allogeneic stem cell transplantation can improve outcomes [113,120,121,122,123].

### 6.6. Chronic Neutrophilic Leukemia

Chronic neutrophilic leukemia (CNL) is a rare myeloproliferative *BCR-ABL* negative leukemia with numerous mature neutrophils [124]. It occurs mainly in older adults and is characterized by marked leukocytosis with neutrophilia and splenomegaly. However, the clinical presentation of CNL may vary from asymptomatic to highly symptomatic with large spleen and constitutional symptoms [125].

The prognosis of CNL is poor, with a median survival of approximately two years [126,127]. Recently, a colony-stimulating factor 3 receptor (*CSF3R*) mutation was identified to drive the disease, and this mutation has been indicated as a criterion for diagnosis of CNL [128]. Very few cases of LC with CNL have been reported in the literature [124,129,130,131,132,133]. Typical manifestations of LC in CNL include erythematous to violaceous papules on varying parts of the body, leukemic vasculitis, gingival hypertrophy, and purpura. Leukemia cutis in CNL should be differentiated from other skin lesions, especially with Sweet syndrome, based on clinical and histological similarities [126,131,132]. However, it can be difficult to differentiate these two diseases due to their clinical and histopathological similarities.

In LC, immature leukocytes infiltrate the skin without significant dermal edema, and skin lesions do not improve with steroids. Although biopsy of Sweet syndrome reveals almost exclusively mature leukocytes, morphology alone may not be sufficient for proper diagnosis in some cases [124]. In some patients, multiple violaceous papules and excoriations have been reported [129]. Nevertheless, it is important to obtain a correct diagnosis of LC as it is associated with a worse prognosis than Sweet syndrome, and the two conditions require different treatments. In Sweet syndrome, improvement is observed after treatment with steroids. In CNL, available therapies include conventional therapy with hydroxyurea as the conventional frontline option and targeted JAK inhibitors. Ruxolitinib has shown significant responses in patients with CNL, but allogeneic stem cell transplantation is the only curative treatment [132]. In patients with LC, CNL is characterized by an aggressive course and short survival [124,133]. However, due to the low prevalence of CNL, the influence of LC remains unclear.

### 6.7. Chronic Myelomonocytic Leukaemia

Chronic myelomonocytic leukemia (CMML) is a hematological malignancy with the characteristics of myelodysplastic syndrome (MDS) and myeloproliferative neoplasms (MPNs), of which incidence has been found to be approximately four cases per 100,000 persons per year [134]. Diagnosis requires the presence of sustained peripheral blood monocytosis (≥1 × 10^9^/L) and bone marrow dysplasia and the exclusion of both myeloproliferative and myelodysplastic neoplasms. In 15–20% of patients, leukemic transformation is observed over three to five years.

Leukemia cutis is a rare event in patients with CMML, with only 89 such cases being recorded thus far in English language publications [135]. In most patients (63%), LC developed after a diagnosis of CMML; however, in one-third of the patients, skin lesions were observed simultaneously with the diagnosis of CMML, and in five (6%), skin lesions were observed several months or years before the development of systemic symptoms. The clinical features include violaceous or red-brown nodules, papules, plaques of varying sizes, and maculo-papular rash. In some cases, painful or pruritic rashes, pustules, and ulcers were observed [135]. The skin changes were localized in any site of the body, most commonly on the trunk and the lower extremities and less frequently in the face, arms, scalp, and neck.

Skin biopsy shows blast cells with granulocytic or monocytic differentiation [136]. Histologically, LC in CMML can include four types of changes based on morphology and immunophenotype [137]. Most commonly, myelomonocytic cells are observed (43%), consisting of CD68- and/or myeloperoxidase-positive myeloid blastic cells, which are also dendritic cell-antigen negative. Less common (38%) is infiltration by CD123-, TCL1- and CD303-positive mature plasmacytoid dendritic cells. Even less common (10%) is blastic plasmacytoid dendritic cell LC, manifesting as monomorphous medium-sized CD4, CD56, CD123, and TCL-1-positive blast cells. Finally, LC can manifest as blastic indeterminate dendritic cell tumors (10%); these are characterized by large blast cells that are positive for CD1a, CD4, CD13, CD33, CD56, and S100 antigens as well as for langerin, CD123, and TCL-1.

Leukemia cutis in CMML is associated with a poor prognosis, especially if extramedullary infiltrations coexist with LC. In several patients, LC proceeds with disease progression, including AML transformation [135,138,139,140,141,142]. The treatment of LC in CMML requires systemic therapy that eradicates the primary underlying disease [143]. Cytoreductive therapy with hydroxyurea or other cytotoxic drugs is indicated in most patients. Treatment with hypomethylating agents such as 5-azacitidine and decitabine can yield overall response rates of 30– 40% and complete response rates of 7% to 17%. Allogeneic stem cell transplant is the only potentially curative treatment; however, it is associated with high morbidity and mortality and is available for only a few patients. Radiotherapy, including total skin electron beam therapy, can be an important part of treatment if LC persists after systemic therapy.

### 6.8. Chronic Myeloid Leukemia

Chronic myeloid leukemia (CML) is a malignant clonal disorder of hematopoietic stem cells. The affected cells are characterized by the rearrangement of t(9;22) (q34;q11), resulting in the formation of a BCR–ABL1 fusion gene. This gene codes for a chimeric BCR-ABL1 protein, which demonstrates high tyrosine kinase activity. The BCR-ABL1 protein has constitutive tyrosine kinase activity, which interferes with the activation of oncogenic cytoplasmic signaling molecules or pathways [144]. While the disease is typically diagnosed in the chronic phase, in 5% to 10% of cases, it may transform to an accelerated or blast phase. In the United States, the incidence of CML in 2022 was estimated at 8860 people [42]. In cases of CML, prognosis has been found to be markedly improved following treatment with various tyrosine kinase inhibitors, including first-generation imatinib, second-generation dasatinib, bosutinib, and nilotinib, and third-generation ponatinib.

Skin lesions in CML may be associated with viral or bacterial infections, treatment with tyrosine kinase inhibitors, and CML leukemic infiltration [145]. However, skin infiltration of CML patients in the chronic phase is extremely rare and is observed in 2 to 8% of patients [3,146]; it more commonly occurs in the blast phase of CML [147,148]. Leukemia cutis has also been reported to be associated with the chronic stable phase of CML, preceding the blast crisis by one week [146]. The patient was treated with leukapheresis and induction chemotherapy with the improvement of skin changes. Subcutaneous nodules with overlying ecchymoses mimicking a neutrophilic panniculitis-like leukemia cutis have also been reported [12,147,148]. Kaddu et al. reported five CML patients with skin infiltration [12]. They identified a variable mixture of mature and immature cells of the granulocytic series (myelocytes, metamyelocytes, eosinophilic metamyelocytes, and neutrophils) or monomorphous mononuclear cells. In other reports, patients with CML developed blastic transformation in the skin [119,149,150].

Singhal et al. described a patient with CML who developed extensive cutaneous manifestations during a second lymphoblastic blast crisis [149]. He presented with multiple subcutaneous skin nodules and extensive violaceous papules and plaques over the limbs and the trunk. The diagnosis was confirmed via fine needle aspiration from the skin nodules, which showed blast with lymphoblastoid characteristics.

Naher et al. describe a patient with CML and AML blastic transformation with multiple cutaneous lesions, diagnosed as LC; the patient had multiple papules, nodules, and plaques of reddish-brown color on his head, neck, back, and chest [150]. In another report, Qi et al. presented the case of a CML patient who developed a skin nodule in the right calf [147]. The diagnosis of CML in the chronic phase was confirmed via PB and BM analysis, whereas the biopsy specimen obtained from the right calf showed an extramedullary myeloid blast crisis of CML despite the chronic phase in the bone marrow.

## 7. Leukemia Cutis in Children

In children, the most common types of leukemia are acute lymphocytic leukemia (ALL) and acute monoblastic leukemia (AML). Children are also more likely to present with LC than adults, with a frequency of approximately 10% in AML and 1–3% in ALL [7,35,151]. A higher prevalence has been reported in infants with congenital leukemia, as noted in 30% of patients [152]. Congenital leukemia is defined as any leukemia diagnosed at birth or in infants under six weeks of age. However, congenital leukemia is extremely rare and accounts for less than 1% of all cases of leukemia in children. The diagnosis of LC in children is often difficult due to its clinical similarity to other dermatologic diseases [107].

Aleukemic LC (ALC) has been also reported in children. It is defined as skin infiltration by a leukemic infiltrate in the absence of BM or PB. Torrelo et al. describe a case of a very aggressive ALC in a newborn, presenting as “blueberry muffin” skin lesions [153]. The child did not develop systemic leukemia but died a few months later due to pulmonary hypertension. In another report on a newborn, Blueberry Muffin syndrome was found to co-occur with AML with adrenal localization and cerebrospinal fluid involvement [154]. Unusually, white blood cell count and bone marrow examination revealed no leukemic blasts in PB or BM; furthermore, the patient underwent spontaneous remission and remained in complete remission at a one-year follow-up [154]. A more recent study by Joshi et al. reported the case of a two-year-old child with a very rare manifestation of relapsed B cell ALL in the form of ALC [90]; LC disappeared after two weeks of treatment, but bone marrow and testicular relapse occurred after two months. Elsewhere, Gru et al. reported three additional cases of ALC in pediatric patients [155]. The ALC was associated with MLL gene rearrangement in two patients, and therapy-related AML ALC was noted in the other.

In a large study, Andriescu et al. reported a case series of 31 patients with pediatric LC. The median age at diagnosis of LC was 26.8 months, ranging from 0 months to 19 years [107]. The most commonly involved sites were the lower extremities and head, and the most common morphologies were erythematous or violaceous nodules and papules.

A study of 54 children with AML by Godínez-Chaparro found that 75.9% of the patients presented at least one dermatosis in the course of the disease [156]. LC was identified in eight patients, including two with congenital LC. In addition, the LC patients tended to be younger than those without LC, and none of the patients presented LC before the diagnosis of AML.

A French study of 438 children with AML diagnosed LC in 24 (5.5%) including three patients with cutaneous relapse of AML [157]. Similar to other studies based on adults, most patients with LC AML had acute monocytic leukemia (FAB type M5) [113,158].

Clinically, 16 patients (67%) presented nodules, two (8%) had papules, two (8%) had both nodules and papules, and four (17%) presented plaques. Children with LC were younger than those without LC (*p* < 0.0001). The median age at diagnosis was 1.2 years among patients with LC, and 8.7 years among those without LC (*p* < 0.0001). Fifteen of 24 (63%) patients were aged under two years. In addition, a normal karyotype was less common among patients with LC (8%) than those without LC (26%; *p* = 0.05). *MLL* rearrangement was the most frequent genetic abnormality in patients with LC, observed in almost half of the patients. Next-generation sequencing found the most frequently mutated genes in LC patients to be *KRAS* (16%), *KIT* (16%), and *PTPN11* (16%). Similar CR rates were noted in LC+ (88%) and LC- (89) patients by the end of the consolidation cycle (*p* = 0.77). However, relapse was observed more often in patients with LC (50%) compared to those without (35%), and OS was shorter in patients with LC, showing that LC has a negative prognostic value in children with AML.

Other data indicates that ALL or lymphoblastic lymphoma (LBL) LC can be an early manifestation of leukemia or lymphoma [159]. In the multicenter trial EORTC 58,881, including 1359 children, 1101 with B-cell ALL and 158 with T-cell ALL, cutaneous involvement was noted in 24 (1.8%) children: 15 with ALL and 9 with LBL. Among the children with LC, 18 belonged to the B lineage: 11 with ALL and 7 with LBL. In the 15 patients with hematologic disease, skin lesions were observed within a median time of six weeks before diagnosis, i.e., from a few days to a maximum of eight months. Most of the patients (21 patients) had at least one skin lesion located on the head, and seven children with high-risk ALL had diffuse cutaneous changes. Induction therapy resulted in complete remission in 23 patients. At the time of publication, 17 of the 24 children were in the first CR, lasting a median of three years (two months to five years), and three were in the second remission, lasting 14 to 24 months.

Several children with ALL and LC demonstrated KMT2A rearrangement (KMT2A-r) [160]. Such children, i.e., with KMT2A-r ALL, unfortunately, have a poor prognosis, with low overall survival and poor outcomes if treated with chemotherapy alone [161].

## 8. Conclusions

Leukemia cutis (LC) is a dermatological manifestation of leukemia characterized by the infiltration of the skin by leukemic cells, which may originate from either myeloid or lymphoid lineages. In most cases, cutaneous lesions manifest after a systemic leukemia diagnosis. However, in some instances, LC is identified concurrently with the detection of generalized leukemia and occasionally even before the hematological diagnosis. The highest incidence of LC is observed in chronic lymphocytic leukemia, monocytic and myelomonocytic acute myeloid leukemia, and T-cell lineage leukemia. Clinical presentations vary in terms of appearance, location, and quantity of leukemic skin infiltrations. A skin biopsy is imperative for distinguishing between malignant infiltration and non-specific changes. The prognosis for patients with LC is contingent on the type of leukemia, involvement of other organs, and response to specific treatments.

## Figures and Tables

**Table 1:** Selected large studies of leukemia cutis.

Authors/Reference	No. of Patients						Clinical Characteristics	Time from Leukemia Diagnosis to LC Development	Survival from LC Diagnosis
	Total	AML	ALL	CML	CLL	Other			
Su et al., 1984 [1]	42	20	3	3	16	0	Multiple papules and nodules (60%), infiltrative plaques (26%), macules, nodules, ulcers	LC after systemic leukemia—23 mo. (55%), before—3 mo. (7%), concomitant 16 mo. (38%)	10–60 mo. (range)
Yook et al., 2022 [3]	56	40	8	3	2	MDS-3	Plaques (28%), papules (27%), patches (18%), nodules (16%)	12.3 mo. (mean)	5.4 mo. (mean)
Kaddu et al., 1999 [12]	26	17	0	9	0	0	Solitary or multiple reddish to violaceous papules, plaques, and nodules (17 pts.) generalized erythematous maculopapular eruption (9 pts.)	0 to 13 mo. in AML pts. (range), 36–72 months (mean of 52.4 mo.) in CML pts.	AML 1–25 mo. (range), CML 3–17 mo. (range)
Chang et al., 2021 [5]	42	24	3	1	1	MDS-8, ALL-5	Papules (38%), nodules (29%), plaques (16%), ulcers (10%)	16.3 mo. (mean)	7.2 mo. (median)
Kang et al., 2013 [13]	75	49	18	7	0	MDS-1	Nodules (33%), papules (30%), and plaques (17%)	16.2 mo. (mean) in 58 pts. after systemic leukemia diagnosis, 2.3 mo. (mean) in 4 pts. LC before systemic leukemia diagnosis, 13 pts. concurrent diagnosis with systemic leukemia	8.3 mo. (median)
Martinez-Leborans et al., 2016 [14]	17	12	0	4	1	0	Nodules (7 pts.), papules (5 pts.), erythematous-violaceous plaques (4 pts.)	aleukemic LC—5 pts.	7 pts. died during the first year
Li et al., 2018 [15]	10	9	0	0	0	CMML-1	Generalized papules or nodules (5 pts), localized masses (5 pts).	4–72 mo. in 7 pts. after systemic leukemia diagnosis	6 pts. died within 2–12 mo.
Watson et al., 2006 [16]	8	5	0	2	1	0	Erythematous papules (75%), nodules and plaques	2–114 mo. (range); 2 pts. at presentation	14 mo. (median) and 3–39 mo. (range)

Abbreviations: ALL—acute lymphoblastic leukemia, AML—acute myeloid leukemia, ATLL—adult T-cell leukemia–lymphoma, CLL—chronic lymphocytic leukemia, CML—chronic myeloid leukemia, CMML—chronic myelomonocytic leukemia, Hb—hemoglobin, LC—leukemia cutis, MDS—myelodysplastic syndrome, mo.—month, pts.—patients.

## Data Availability

The data presented in this study are available in this article.
